# Uncommon cavernous malformation of the optic chiasm: a case report

**DOI:** 10.1186/2047-783X-17-24

**Published:** 2012-08-14

**Authors:** Xianbin Ning, Kan Xu, Qi Luo, Limei Qu, Jinlu Yu

**Affiliations:** 1 Department of Neurosurgery, First Hospital of Jilin University, 71 Xinmin Avenue, Changchun, 130021, People’s Republic of China; 2 Department of Neurosurgery, First Hospital of Beihua University, 12 Jiefang Avenue, Jilin, 132011, People’s Republic of China; 3 Department of Pathology, First Hospital of Jilin University, 71 Xinmin Avenue, Changchun, 130021, People’s Republic of China

**Keywords:** Optic chiasm, Cavernous malformation, Stroke

## Abstract

Cavernous malformation (CM) is a vascular malformation disorder characterized by a berry-like mass of expanded blood vessels. CM, originating from the optic chiasm. usually leads to chiasma syndrome presenting with bitemporal hemianopsia. We report a 28-year-old male presenting with left homonymous hemianopsia. Magnetic resonance imaging (MRI) revealed an occupied lesion located in the right side of the optic chiasm, and a clinical diagnosis of chiasmal CM was made. Microsurgical excision was performed *via* anterolateral pterional craniotomy. The patient showed good recovery with slight improvement of the visual field deficits after the operation. No CM recurrence was discovered during the follow-up MRI scans.

## Background

Intracranial cavernous malformations (CMs) are not uncommon in the clinic and account for 10 to 20% of intracranial vascular diseases [1]. CMs are usually found in the brain parenchyma, and CMs of the optic chiasm are extremely rare, accounting for <1% of intracranial CMs [2]. The clinical symptoms of chiasmal CMs depend on the lesion size and amount of bleeding. If the CM is large or the volume of bleeding is high, then the chiasmal CM usually elicits stroke symptoms (that is, headache, vision loss and visual field defects) [3,4]. CMs involving the optic chiasm typically cause bilateral temporal visual field defects [5,6]. Here, we describe a case with a CM located on the right side of the optic chiasm, in which the patient presented with bilateral left homonymous hemianopsia in the visual field examination. After definitive diagnosis, the CM was removed surgically with a satisfactory outcome. We further review pertinent literature on the clinical and radiological features and surgical treatment of CMs.

## Case presentation

A 28-year-old male complained of blurred vision in his right eye, which started 2 months before presentation and had worsened about 10 days before. He was otherwise healthy and had no negative past medical history or history of ocular trauma or surgery. Two months before presentation, the patient visited the Department of Ophthalmology, First Hospital of Jilin University, due to a sudden onset of blurred vision. Eye examination showed no obvious abnormalities, and no exact diagnosis was made. The patient did not return to the clinic until the sudden worsening of blurred vision with forehead pain 10 days before presentation. His best-corrected visual acuity was 0.5 in the right eye and 0.4 in the left eye. Anterior segment slit-lamp examination showed no obvious abnormalities. Fundus examination revealed binocular mild primary optic atrophy. Visual field examination showed left homonymous hemianopsia. No other neurological abnormalities were found. No abnormalities were found in the routine analysis of blood, blood coagulation or pituitary hormones.

**Figure 1:**
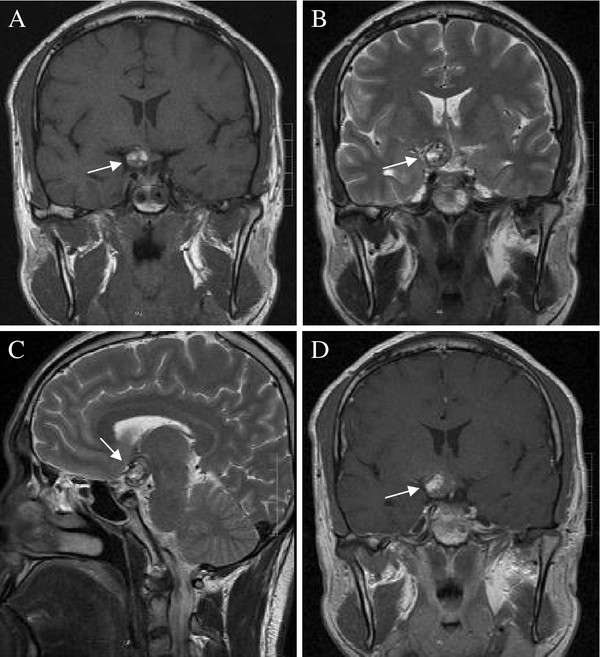
**Magnetic resonance images of the cavernous malformation of the optic chiasm (arrows).** (**A**) The mass lesion demonstrated diffuse hyperintensity on T1-weighted imaging and mixed signal intensity; (**B-C**) The lesion was surrounded by a marked hypointense peripheral rim of hemosiderin on T2-weighted imaging. (**D**) No significant contrast enhancement was observed after the enhanced scanning.

Magnetic resonance imaging (MRI) demonstrated a 1.8 mm × 1.7 mm mass located in the right suprasellar cistern and the right side of the optic chiasm. The mass lesion had clear boundaries and no edema around the lesion. T1- and T2-weighted images showed mixed signal intensity surrounded by a peripheral rim of low signal on the T2-weighted images, which indicated that the right side of the optic chiasm was pressed. Enhanced scans showed no significant enhancement (Figure 1). A diagnosis of chiasmal CM was made.

Consequently, after approval by the medical ethics committee and provision of informed consent by the patient, the patient was treated with microsurgical dissection *via* right pterional craniotomy. The CM was protruded above the optic chiasm at a depth of 5 mm, and skewed to the right. The CM was cystic, containing a lot of dark blood. The substantial part of the CM had a tough texture and moderate blood supply, and it had a close adhesion with the optic nerve. We completely removed the CM between the bilateral optic nerves, and the optic chiasm was retained intact.

**Figure 2:**
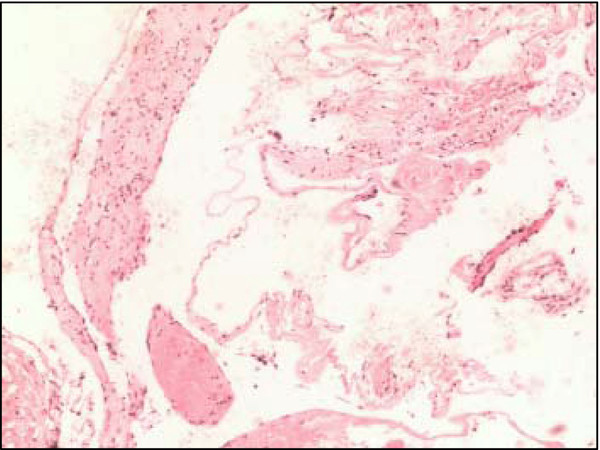
**Pathological results of the cavernous malformation.** Photomicrograph showed thin-walled cavernous vascular spaces with little intervening brain (HE × 200).

**Figure 3:**
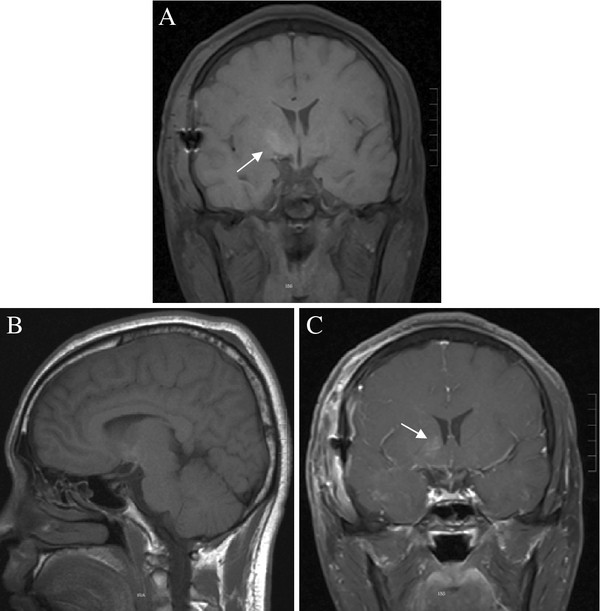
**Magnetic resonance images of postoperative follow-up.** (**A,B**) MRI scanning and (**C**) enhanced scanning revealed the complete removal of chiasmal cavernous malformations and no recurrence, but a hyperintensity in the basal ganglia was found (arrows).

Postoperatively, the headache was reduced and the patient reported no further visual acuity or field deficits. Histological examination revealed that the lesion consisted of thin-walled cavernous cavities with visible separations, with no nerve tissue among them. A definite diagnosis of chiasmal CM was made (Figure 2). The six-month follow-up showed that his best-corrected visual acuity was 0.8 in both eyes, the headache had disappeared, visual field deficits were slightly improved, and no CM recurrence was found by an MRI scan. However, a hyperintensity in the basal ganglia was found (Figure 3).

## Discussion

Intracranial CMs are diagnosed as supratentorial (80%), infratentorial (15%), at the spinal cord (5%) and, rarely, at the optic chiasm (<1%) [7-9]. However, chiasmal CM patients are prone to bleeding and stroke, which involves the nerve fibers of both eyes and presents with chiasma syndrome [10-12]. Typical optic chiasm syndrome after stroke involves the sudden onset of postorbital and frontal pain, sudden vision loss and bilateral temporal visual field defects (first proposed in 1982 by Maitland) [11]. These symptoms have been verified in case series of chiasmal CMs by Shibuya [10] (14 cases, in 1995) and by Crocker [7] (32 cases, in 2008). Here, we report a chiasmal CM in a patient presenting with vision loss and headache after stroke. Especially, visual field examination of this patient revealed bilateral left homonymous hemianopsia that did not meet the typical visual field changes associated with optic chiasm syndrome after stroke, because MRI revealed an occupied lesion located in the right side of the optic chiasm. Because the optic chiasm is a small and symmetrical structure, the whole optic chiasm usually is involved when chiasmal CM occurs here, and the optic chiasm syndrome is very typical after chiasmal apoplexy. But in this report, the CM located in one side of the optic chiasm and the symptoms were typical except for the visual field changes, which make the case exceptional. We are interested in reporting this case to enrich the understanding of chiasmal CMs.

MRI is currently considered to be the preferred imaging technique for the detection and characterization of CMs, especially atypical CM cases. As early as 1999, Arrué [13] reported that MRI could clearly reflect the different bleeding periods of CMs, such as the acute and subacute phases. They also found that the center of CMs was methemoglobin surrounded by a hemosiderin ring, also called the “iron ring sign”, which showed mild or no enhancement after enhanced MRI scan. Cases reported in the literature afterwards, including this case, were consistent with these imaging features [2,14,15]. According to previous reports, in MRI, chiasmal CMs usually are located in the middle of the optic chiasm [7]. However, the case in the present report was one-sided, which enriched the MRI of the optic chiasmal CMs. This finding indicates that a one-sided CM of the optic chiasm should also be a potentially anticipated finding.

The differential diagnosis of chiasmal CMs mainly includes some tumors in the optic chiasm, such as glioma, granuloma, metastatic tumors and germ cell tumors [16-19]. These lesions can cause enlargement of the optic nerve or chiasm but usually do not exhibit signs of hemorrhage. Intense enhancement after contrast administration is common for these lesions. Besides, the “iron ring sign” of chiasmal CMs should distinguish chiasmal CMs from other tumors. A retrospective analysis of the MRI results in the present case showed that the CM was located in the optic chiasm. It presented a mixed signal on T1- and T2-weighted imaging, peripheral rim of hemosiderin on T2-weighted imaging, and no enhancement after enhanced MRI, consistent with the MRI features of CMs, although its location was different from that of previously reported CMs.

Once a definite diagnosis of chiasmal CM is made, surgical resection should be performed to relieve the visual impairment induced by optic chiasm syndrome. In general, surgical approaches include frontotemporal, orbital zygomatic approach, pterional and interhemispheric approaches. The choice of surgical approach depends on the lesion size, disease development and familiarity of the surgeon with specific surgical approaches [2,10,14,15]. Liu *et al.*[2] reported the surgical resectioning of 65 cases of CMs from the optic pathway in a retrospective analysis, and found that 76% of cases were treated with the anterior approach, 16% with the interhemispheric approach and 8% with another approach. In the present case report, we removed the chiasmal CM by using the frontotemporal approach. During surgery, the anterior gap and side of the optic chiasm were successively exposed after lifting the frontal base and opening the sylvian fissure, respectively. The fully exposed surgical field was conducive to the complete removal of the CM.

Not all chiasmal CMs can be thoroughly removed by surgical treatment. For example, the total resection rate of 65 cases reported by Liu *et al.*[2] was 60%, and optic nerve decompression was necessary in some cases. Although partial resection of the CM by releasing the hematoma after stroke contributes to the improvement of visual acuity, the current gold standard for the treatment of chiasmal CMs is complete surgical removal. Reasons for why some chiasmal CMs cannot be totally removed may include the limited space of the optic chiasm, tight adhesion to the optic nerve and extreme surgical difficulty, such as when the bleeding of the CM is light and there is no obvious hemosiderosis around the CM, which can lead to an unclear boundary [20,21]. The development of MRI particularly contributes to the increased diagnosis and complete surgical removal rate of chiasmal CMs [13,22]. As demonstrated in our case, under the correct guidance of MRI, the chiasmal CM was safely and completely resected with preservation of good cranial nerve function. At the six-month follow-up of MRI, a hyperintensity was found in the basal ganglia. The abnormal signal was thought to be caused by an intraoperative traction injury during removal of a neighboring CM because the mass protruded into the right suprasellar cistern from the right side of the optic chiasm. Fortunately, the patient had no related symptoms.

## Conclusions

In summary, chiasmal CMs usually present with typical optic chiasm syndrome in most clinical cases, but visual field changes may occur when the chiasmal CM is located at the side of the optic chiasm. In either case, definite preoperative diagnosis by means of MRI technology and complete surgical removal by selecting the appropriate surgical approach contribute to satisfactory outcomes.

## Consent

Written informed consent was obtained from the patient for publication of this case report and any accompanying images. A copy of the written consent is available for review by the Editor-in-Chief of this journal.

## Abbreviations

CM: Cavernous malformation; MRI: Magnetic resonance imaging.

## Competing interests

The authors declare that they have no competing interests.

## Authors’ contributions

XBN wrote the initial draft. KX and QL analyzed and interpreted the patient data. LMQ performed the histological examination. JLY was the surgeon and a major contributor in designing the manuscript. XBN and KX contributed equally to this work. All authors read and approved the final manuscript.

## Authors’ information

Xianbin Ning has a master’s degree; Kan Xu has a doctor’s degree; Luo Qi has a doctor’s degree; Limei Qu has a bachelor’s degree; Jinlu Yu is a neurosurgeon and has a doctor’s degree.
